# Research on Wearable Devices for Pedestrian Navigation Based on the Informer Model Zero-Velocity Update Architecture

**DOI:** 10.3390/s25082587

**Published:** 2025-04-19

**Authors:** Shuai Zhang, Haotian Gao, Fushengong Yang

**Affiliations:** School of Art and Design, Shenyang Ligong University, Shenyang 110159, China; 15840280722@163.com (H.G.); 15640435051@163.com (F.Y.)

**Keywords:** wearable inertial measurement unit, zero-velocity update, self-adaptive, wearable technology

## Abstract

When natural disasters such as earthquakes occur, accurate navigation and positioning information may not be available, making a purely inertial pedestrian navigation system particularly important for rescuers. In this paper, researchers propose a zero-velocity update architecture for pedestrian navigation based on the Informer model, which is integrated into wearable devices. This architecture modifies the fully connected layer of the Informer model to be used for the binary classification task of the zero-velocity update method (ZUPT), allowing for accurate identification of gait information at each moment using only inertial measurement data. By wearing the device on the foot during natural disasters like earthquakes, the location of the pedestrian can be more accurately determined, facilitating rescue efforts. During the experimental process, a Kalman filter model was constructed to achieve zero-velocity updating of the pedestrian’s motion trajectory. A 2000 m walking experiment and a 210 m mixed-gait experiment were conducted to accurately identify gait information at each moment, thereby reducing the cumulative error of the inertial system. Subsequently, a convolutional neural network (CNN) model and a model combining CNN with a long short-term memory network (CNN + LSTM) were introduced as comparative experiments to verify the performance of the proposed architecture. The experimental results demonstrate that the proposed architecture enhances the adaptability of the zero-velocity update algorithm in underground or sheltered spaces, with all results outperforming the other two models.

## 1. Introduction

In natural disasters and production safety accidents, such as earthquakes and mine collapses, it is often necessary to navigate through complex underground or obscured spaces. This makes it increasingly difficult for rescuers to carry out their operations and can even endanger their lives [1]. Therefore, accurately determining the location of trapped individuals is crucial during rescue operations, as it can significantly reduce the time required to rescue survivors and increase the success rate. While global navigation satellite systems (GNSSs) can provide clear location information in open areas, they are unable to function continuously in narrow or obscured spaces, making the accurate positioning of survivors particularly challenging in these environments. Hence, achieving precise positioning of survivors in such spaces is of utmost importance. Due to the limitations of GNSS in underground or sheltered spaces, researchers both domestically and internationally have conducted extensive studies on various systems, including pure inertial measurement unit (IMU) guidance systems [2], Ultra-Wide-Band (UWB) systems [3], visual aids [4], and indoor maps [5]. However, these methods have certain limitations. For instance, UWB systems require prior installation in the environment, visual aids depend on computationally intensive image processing systems that are susceptible to environmental changes, and indoor maps necessitate prior acquisition of environmental information, Therefore, this research focuses on developing a wearable device based on a pure IMU navigation system, which can provide valuable time for rescuing survivors in narrow or obscured spaces during natural disasters.

With the development of micro-electromechanical systems (MEMSs), miniaturized, low-power, and low-cost IMUs have been widely integrated into mobile terminals and smart wearable devices [6]. Due to the characteristics of IMUs, navigation errors accumulate over time and distance [7]. The progress in MEMS sensor technology in size, cost, weight, and power consumption allows for new research opportunities in the navigation field [8]. Commonly, IMUs are placed on the foot of pedestrians to successfully conduct pedestrian navigation to estimate position, velocity, and orientation [9,10]. To reduce the errors in pedestrian navigation systems, the ZUPT is widely applied [11]. The Kalman filter-based implementation of the ZUPT is able to correct not only velocity, position, and attitude errors, but also most of the position drift under motion gaits [12]. In the ZUPT method, the pedestrian gait cycle is often divided into a standing posture and a swing phase [13]. If a pedestrian is in a standing posture, his feet would touch the ground and remain stationary for a short period of time. It is in the standing posture that the ZUPT intervals occur. The swing phase is when the pedestrian’s feet fail to touch the ground for a longer period of time. It is in the swing phase that the non-ZUPT intervals occur. In order to obtain results that better match the actual trajectory of pedestrians, the standing phase of each gait cycle must be accurately detected [14]. Conventional ZUPTs usually adopt the fixed-threshold method to constrain the inertial measurement data by setting thresholds in advance. However, conventional ZUPTs have a limited range of motion and can only provide the closest true zero-velocity detection for uniform motion types, which cannot accurately determine the gait of pedestrians under different road conditions [15]. When rescuers are under the ground, in sheltered spaces with unknown conditions, and in complex environments, the identification of the zero-velocity phase of the gait cycle becomes difficult. Under these circumstances, rescuers are very likely to change their motion types, so the fixed-threshold method detection is prone to failure, thereby resulting in greater deviations in the estimated pedestrian trajectories.

Many advances have been made by previous researchers in the field of improving traditional fixed-threshold-based zero-velocity update architectures for pedestrian navigation. Johan et al. [16] put forward a Bayesian zero-speed detector for a foot-operated inertial navigation system and an adaptive thresholding approach, which can select a separate threshold for each speed or motion pattern. However, the system can only be applied to motion states, including walking and fast walking. Cho et al. [17] proposed a threshold-free zero-velocity detection algorithm by identifying the shape of a simplified signal to detect the zero-velocity threshold, thereby employing accelerometer signals to detect the most likely location of the zero-velocity update. Similarly, the threshold-free zero-velocity detection algorithm is only applicable to the states of walking and fast walking. In addition, Ju et al. [18] came up with a dual-IMU Pedestrian Dead Reckoning (PDR) system to improve performance, which was compared to traditional algorithms by imposing joint constraints between the two sensors. Zhang et al. [19] developed a new hybrid framework that integrated a ZUPT-triggered algorithm, a zero-angular-rate update (ZARU) algorithm, a state lock foot state classifier, a magnetic interference detector, a human-motion-classifier-assisted adaptive fusion module (AFM), and an error-state Kalman filter (ESKF). Dai et al. [20] presented an inertial pedestrian navigation algorithm based on zero-velocity updates and posture self-observations, which adopted a four-conditional zero-velocity detection algorithm to achieve the correction of navigation errors. However, the flexibility and generalization capability of the above methods still require further improvement.

Deep learning has become a mainstream technology in the field of neural networks because of their back propagation algorithmic automatic learning feature [21]. The application of deep learning models can realize the dynamic adjustment of thresholds and correction coefficients for ZUPT systems. Among the deep learning models, the CNN [22] and LSTM [23] are the most popular in the field of inertial navigation. Yu et al. [24] offered a zero-velocity detection method based on a CNN network, which could work better with pedestrians with different physical characteristics and walking patterns when compared to the fixed-threshold method. Wagstaff et al. [25] proposed an LSTM-based zero-velocity detector, which was more accurate in detecting motion states such as walking, running, and stair-climbing when compared to the Stance Hypothesis Optimal Estimation (SHOE) and Angular Rate Energy Detector (ARED).

In recent years, the emergence of various NLP (Natural Language Processing) models [26] has promoted the field of deep learning, and the Informer model was designed to specifically solve the problem of temporal prediction [27], which has attracted much attention from the research field. Traditional CNN models and LSTM models are not well suited to long-sequence data [28] and can only extract information in the local area of the inertial measurement data. The Informer model aims to solve the problem of temporal sequence prediction with the ‘ProbSparse’ attention mechanism. In this research, the fully connected layer of the Informer model was improved to better accommodate pedestrian navigation systems by adding Sigmoid activation functions.

The first part introduces the research objectives, and the second part of this research introduces the zero-velocity update architecture for pedestrian navigation and describes the feature label selection method. The third part of this research presents the improved Informer model and its evaluation metrics. The fourth part of this paper describes the design process as well as the experimental process, including data acquisition, model training, and navigation trajectory solving. The fifth part is the summary of the whole paper.

## 2. Pedestrian Navigation Zero-Velocity Update Architecture

**Figure 1:**
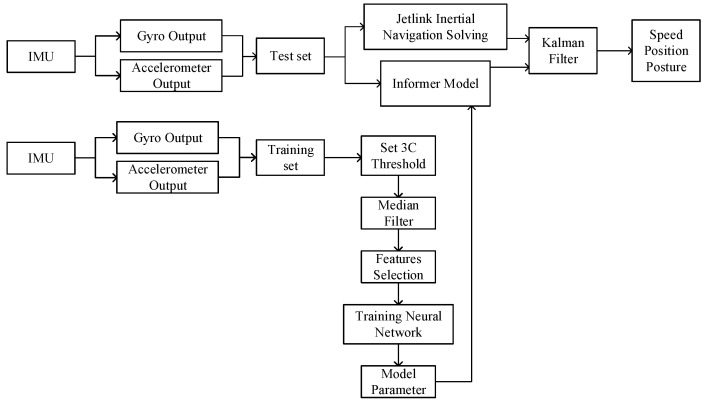
Pedestrian zero-velocity update architecture.

The zero-velocity update algorithm is designed to suppress the divergence of position and velocity errors by applying a Kalman filter to estimate the navigation errors within the zero-velocity intervals during the movement states of pedestrians [29]. The pedestrian zero-velocity update architecture proposed in this research is based on the traditional zero-velocity update algorithm with fixed thresholds [14]. The Informer model was introduced into the zero-velocity detector, which improves the ability to identify different movement states by training the neural network. The pedestrian zero-velocity update architecture based on the Informer model is shown in Figure 1.

In the pedestrian zero-velocity update architecture proposed in this research, firstly, the six-dimensional inertial measurement data (three-axis gyroscope and three-axis accelerometer) of each motion state were collected in advance to serve as the training set, and the zero-velocity intervals output by the 3C method were used as the feature label. Since the step length and motion characteristics of pedestrians with different physiques were not the same, the selection of the feature labels required adjusting the three discriminant condition thresholds in the 3C method. The process of adjusting the thresholds generated unwanted small burr signals, so the output in the 3C method needed to be filtered by the median to acquire more suitable feature labels. Then, the six-dimensional inertial measurement data and one-dimensional feature labels were integrated into the seven-dimensional training set. Later, the seven-dimensional training set data with the improved Informer model were trained, and the trained model parameters were saved.

In this research, the inertial measurement data of the pedestrians to be tested served as the test set and output for the trained Informer model. Then, the trained Informer model was used to predict the inertial measurement data and adaptively output the appropriate ZUPT sequence. Finally, the errors were corrected by the ZUPT sequence and a Kalman filter to achieve zero-velocity updates of the pure inertial pedestrian navigation system.

### 2.1. Selection of Zero-Velocity Intervals

**Figure 2:**
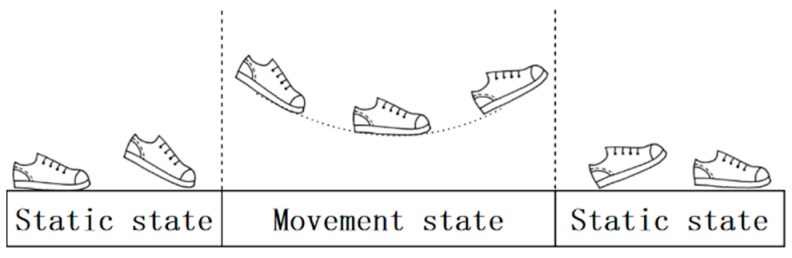
Pedestrian gait cycle diagram.

The gait cycle of pedestrian motion is divided into two states: the static state and the movement state. In addition, the question of how to accurately determine the intervals of the resting gait is the focus of this research. The pedestrian gait cycle is shown in Figure 2.

The IMU is placed on the foot of the pedestrian after the pedestrian completes a complete gait cycle while the pedestrian is in a static state. ZUPT intervals can be obtained by analyzing the data characteristics of the gyroscope and accelerometer. The ZUPT intervals under the pedestrian gait cycle can be obtained with data from the gyroscope or accelerometer, respectively, or from a linear combination of data from both sensors [30]. In this research, the ZUPT intervals were determined by the agency of a three-condition (C1, C2, and C3) judgment algorithm [14]. C1 represents the judgment of the acceleration amplitude. C2 represents the judgment of the acceleration variance, and C3 represents the judgment of the angular velocity amplitude. To judge the accelerometer output synthesis amplitude, the C1 condition can classify the human movement state into two categories: a static state and a movement state. When the accelerometer output synthesis amplitude is between the given upper and lower thresholds, the pedestrian’s body is stationary, which means he is at rest. In addition, the output value of C1 is set to ‘1’. If the amplitude is not in this range, the output is set to ‘0’. The equation for calculating the acceleration amplitude is as follows:

(1) $$\left|\alpha_{k}\right|=\sqrt{\alpha_{k}{\left(x\right)}^{2}+\alpha_{k}{\left(y\right)}^{2}+\alpha_{k}{\left(z\right)}^{2}}$$

where $\alpha_{k}$ is the acceleration value at moment k.

C2 determines the local variance of the accelerometer to obtain its output. If the local variance of the accelerometer output is lower than the given threshold, the pedestrian’s body is stationary. The output value is set to ‘1’. If the local variance is not in this range, the output value is set to ‘0’. The acceleration variance calculation equation is as follows:

(2) $$\sigma_{\alpha_{k}}^{2}=\frac{1}{2s+1}\sum_{q=k-s}^{k+s}{\left(\alpha_{q}-{\bar{\alpha }}_{k}\right)}^{2}$$

in which

(3) $${\bar{\alpha }}_{k}=\frac{1}{2s+1}\sum_{q=k-s}^{k+s}\alpha_{q}$$

where s is the number of half-window samples.

C3 is used by judging the synthesis of the amplitude of the gyroscope output. If the angular velocity synthesis amplitude is lower than a given threshold, the pedestrian’s body is stationary. The equation is shown below.

(4) $$\left|\omega_{k}\right|=\sqrt{\omega_{k}{\left(x\right)}^{2}+\omega_{k}{\left(y\right)}^{2}+\omega_{k}{\left(z\right)}^{2}}$$

where $\omega_{k}$ is the angular velocity value at moment k.

The gait is considered stationary only if the above three conditions are met simultaneously. In this research, the output 0–1 sequence of the 3C method was used as the feature label, which was integrated with the six-dimensional inertial measurement data to obtain the seven-dimensional training set and test set data.

### 2.2. Jet-Linked Inertial Navigation Equations

The inertial measurements placed on the foot are not directly available for pedestrian navigation and must be solved for navigation. The jet-linked inertial navigation update equations are mainly the attitude update, the velocity update, and the position update [31]. The attitude update differential equation is shown as follows:

(5) $${\dot{C}}_{b}^{n}=C_{b}^{n}(\omega_{nb}^{b}\times )$$

where $C_{b}^{n}$ is the attitude matrix of the body relative to the navigation coordinate system, and $\omega_{nb}^{b}$ is the angular velocity of the gyroscope output.

The navigation coordinate system (n) typically represents a geographic coordinate system, where the x-axis points towards the east direction, the y-axis points towards the north direction, and the z-axis points vertically upward. The body coordinate system (b) is usually a coordinate system that is rigidly attached to the carrier. Its axes are typically aligned with the carrier’s motion direction, with the x-axis pointing along the carrier’s forward direction, the y-axis pointing along the carrier’s left–right direction, and the z-axis pointing along the carrier’s up–down direction.

Since the accelerometer measures the specific force, the acceleration of the carrier itself needs to subtract the Earth’s gravity component from the specific force, i.e.,

(6) $$a^{n}(t)=f^{n}(t)-g^{n}$$

where $g^{n}=\left[0\;0\;g\right]$. g is the local gravitational acceleration. Thus, the velocity update equation is

(7) $$v^{n}(t)=v^{n}(0)+\int a^{n}(t)dt$$

where $v^{n}(0)$ is the initial velocity. Hence, the position update is obtained as

(8) $$s^{n}(t)=s^{n}(0)+\int v^{n}(t)dt$$

where $s^{n}(0)$ is the initial position. The ‘east–north–sky’ geographic coordinate system is chosen as the navigation coordinate system. Because the speed of the pedestrian movement state is slow, combined with the characteristics of MEMS devices, the inertial navigation error equation can be simplified as follows:

ϕ (Attitude Error) describes the deviation in the vehicle’s attitude, which affects navigation accuracy. *V* (Velocity Error) describes the deviation in the vehicle’s velocity, which affects the accumulation of position errors. *P* (Position Error) describes the deviation in the vehicle’s position, which is the primary performance metric of a navigation system.

(9) $$\begin{array}{c} \delta \dot{\phi }=-C_{b}^{n}\varepsilon^{b}\\ \delta \dot{V}=C_{b}^{n}f^{b}\times \delta \phi^{n}+C_{b}^{n}\nabla^{b}\\ \delta \dot{P}=\delta V^{n} \end{array}$$

where $\nabla^{b}$ is the constant zero offset of the accelerometer, and $\varepsilon^{b}$ is the random constant drift of the gyroscope. The zero offset of the accelerometer and gyroscope is as shown in Table 1.

### 2.3. Kalman Filtering Method

Kalman filtering is a widely adopted data optimization method in the industry [32]. In inertial navigation systems, it is used to address the problem that navigation errors are bound to accumulate over time. Current related research commonly adopts Kalman filtering to optimize navigation data. The zero-velocity update architecture proposed in this research used the Kalman filter to correct the navigation errors, and the Kalman filtering method consists of two processes: the state estimation and the state update [33]. The state estimation estimates the current moment state based on the previous moment state. The update process obtains the optimal state by combining the predicted current moment state with the observed state. The system measurement equation is shown below:

(10) $$\begin{array}{c} \dot{X}=\Phi_{k,k-1}X+W\\ Z=HX+V \end{array}$$

where Φ is the state transfer matrix, W is the system noise vector, and V is the measurement noise vector. In $X=(\delta \phi ,\delta V,\delta P,\nabla^{b},\varepsilon^{b})$, the state vectors are attitude, velocity, position, accelerometer zero bias, and gyroscope zero bias. The observation matrix H is

(11) $$H=\left[0_{3\times 3}I_{3\times 3}0_{3\times 3}0_{3\times 3}\right]$$

The process of Kalman filtering is as follows:

1. State one prediction:

(12) $${\dot{X}}_{k,k-1}=\Phi_{k,k-1}{\dot{X}}_{k-1}$$

2. One-step prediction mean square error equation:

The role of Q is to characterize the intensity and correlation of the system process noise, and it is an essential parameter in the Kalman filter for updating the error-state estimates.

(13) $$P_{k,k-1}=\Phi_{k,k-1}P_{k-1}\Phi_{k,k-1}^{T}+Q_{k-1}$$

where P is the covariance array of the measurement noise.

3. Kalman filtering gain filtering:

(14) $$K_{k}=P_{k,k-1}H_{k}^{T}{\left(H_{k}P_{k,k-1}H_{k}^{T}+R_{k}\right)}^{-1}$$

4. State estimation equation:

(15) $${\dot{X}}_{k}={\dot{X}}_{k,k-1}+K_{k}(Z_{k}-H_{k}{\dot{X}}_{k,k-1})$$

5. Estimated mean square error equation:

(16) $$P_{k}=(I-K_{k}H_{k})P_{k,k-1}{\left(I-K_{k}H_{k}\right)}^{T}+K_{k}R_{k}K_{k}^{T}$$

## 3. Zero-Velocity Detection Algorithm

### 3.1. Informer Neural Network

The Informer model adopts the attention mechanism of ‘ProbSparse’ self-attention, which can significantly reduce the computation and storage space while ensuring the accuracy of the model, and it is mainly used for the prediction of time series. The model adopts a complete encoder–decoder architecture, which mainly consists of two parts: the encoder and the decoder. The encoder can extract feature information from time-series data and has good generalization ability and robustness. When the Informer’s encoder is used for zero-velocity detection binary classification tasks, it can classify the data in inertial measurements into stationary or motion state data, thus reducing the interference of the operational dynamic data with the system and improving the accuracy and stability of the positioning and navigation.

**Figure 3:**
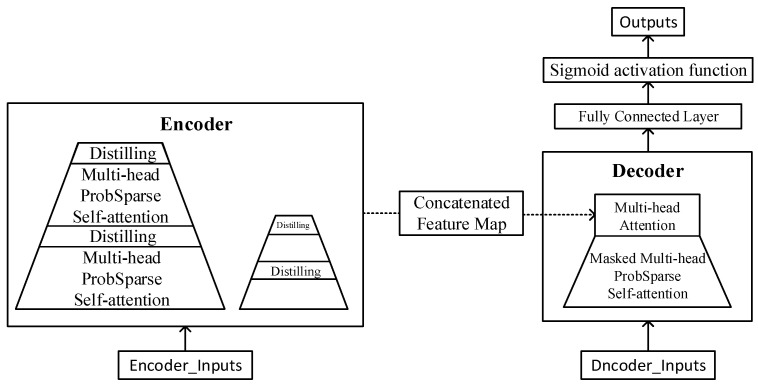
Informer neural network structure diagram.

The zero-velocity update architecture proposed in this research not only improves the fully connected layer, but also improves the encoding method to make it better adapted to the prediction of ZUPT intervals. The model was trained on seven-dimensional training set data (six-dimensional inertial measurement data and a one-dimensional three-conditional ‘with’, followed by long-sequence label data). The improved model is shown in Figure 3.

The encoder in the Informer model is designed to extract robust long-term dependencies in long-range sequence inputs. Since the position relationship of the data is particularly important for the time-series prediction problem, the Informer model needs to encode the position of the inertial measurement data to enhance its applicability to time-series data sequences, which further enables the model to predict longer sequences. The positioning encoding formula is as follows:

(17) $$\begin{array}{c} Pos\_Enc(pos,2i)=sin(pos/10000^{2i/d_{model}})\\ Pos\_Enc(pos,2i+1)=cos(pos/10000^{2i/d_{model}}) \end{array}$$

where pos is the location, and $i\in (0,1,\ldots ,d_{model}/2)$ is the dimensionality. The self-contained temporal encoding method may require complex conversion and processing to adapt to the input format of the neural network. Therefore, in this research, the researchers used ordinal encoding instead of traditional temporal encoding, which does not require additional preprocessing and can be computed directly in the neural network, thus reducing the computational effort of the model. In the traditional multi-headed attention scoring mechanism, the attention scoring function adopts a scaled dot product, i.e.,

(18) $$(Q,K,V)=Softmax\left(\frac{QK^{T}}{\sqrt{d}}\right)V$$

where $Q\in R^{L_{Q}\times d}$, $K\in R^{L_{K}\times d}$, $V\in R^{L_{V}\times d}$, and d are the input dimensions. In order to measure the Qurry sparsity, the KL scatter was applied to obtain the sparsity measure equation for the *i*th Qurry.

(19) $$(q_{i},K)=ln\sum_{j=1}^{K_{k}}e^{\frac{q_{i}k_{j}^{T}}{\sqrt{d}}}-\frac{1}{L_{K}}\sum_{j=1}^{L_{K}}\frac{q_{i}K_{j}^{T}}{\sqrt{d}}$$

In turn, the ‘ProbSparse’ self-attribution equation is obtained:

(20) $$A(Q,K,V)=Softmax\left(\frac{\bar{Q}K^{T}}{\sqrt{d}}\right)V$$

where Q, K, and V are the query vector sequence, key vector sequence, and value vector sequence after linear transformation, respectively. And $\bar{Q}$ is the result of Q sparsification.

After the ‘ProbSparse’ self-attentive mechanism is applied, there are redundant V values on the encoder’s feature map. In addition, the ‘distillation’ mechanism compresses the feature dimension by halving the cascade layer input to highlight the dominant features and form a focused self-attentive feature map in the next layer.

(21) $$X_{j+1}^{t}=MaxPool(ELU(Convld({\left[X_{j}^{t}\right]}_{AB})))$$

where ${\left[\cdot \right]}_{AB}$ symbolizes the attention block. Convld(⋅) is the one-dimensional convolution operation, and ELU(⋅) stands for the activation function.

The decoder of the model employs a forward process to produce a long-sequence output. Its internal composition consists of two multi-headed attention mechanisms, where the input data first pass through a masked multi-headed ‘ProbSpare’ self-attention mechanism, which introduces the masked self-attention into a sparse attention mechanism, thus avoiding autoregression. In addition, the data pass through another multi-headed attention mechanism with the output of the encoder. The output of the encoder is given to the fully connected layer. The fully connected layer is usually used for the linear transformation and dimensionality reduction of the input features to achieve the fusion and extraction of features. In this experiment, the encoder input is the seven-dimensional data and the hidden representation of the data output. The decoder input is the inertial measurement data with temporal features and the hidden representation of the label and encoder output.

In the Informer model, the fully connected layer converts the output of the last layer of the model into a prediction of the time series. The nature of predicting the ZUPT is a binary classification problem, so the output of the fully connected layer needs to be changed to a binary classification output, which is trained through a cross-entropy loss function. To verify the degree of inconsistency between the predicted and real values of the model, the loss function (loss function) adopts the form of cross-entropy. Its equation is shown below.

(22) $$L=-\frac{1}{N}\sum_{i=1}^{M}y_{i}log(p(y_{i}))$$

where N is the number of samples. $y_{i}$ is the sign function (0 or 1). $p(y_{i})$ is the predicted probability of $y_{i}$.

In this research, the output of the fully connected layer was mapped to a binary output space for the purpose of classification. First, the output size of the fully connected layer was set to 2 and activated using the Sigmoid activation function. Then, the output was transformed into a probability distribution representing two categories. Then, a cross-entropy loss function was defined, and the model was trained with an optimizer. After the training was completed, the output values of the model were used for the prediction of the binary classification task, and the category with the highest probability value was selected as the prediction result. In this research, the original IMU data with 0–1 labels were trained and predicted by modifying the Informer model, and the final output was set to 1 for zero-velocity intervals that met the ZUPT condition and 0 for non-zero-velocity intervals that did not meet the ZUPT condition. The Sigmoid activation function is

(23) $$S\left(x\right)=\frac{1}{1+e^{-x}}$$

When x→∞, $S\left(x\right)\to 1$. When x→−∞, $S\left(x\right)\to 0$.

### 3.2. Model Evaluation Metrics

Accuracy and recall are two metrics that are widely used in the fields of information retrieval and statistical classification to evaluate the quality of results. Accuracy is employed as the ratio between the number of ‘0’ or ‘1’ features detected and the number of all features detected. Recall is the ratio between the number of ‘0’ or ‘1’ features and the number of all features of the same type in the dataset. To verify the accuracy of the neural network model for the dichotomous classification task, this research used the F1-composite evaluation metric (F1-Measure), which is formulated as follows:

(24) $$F1=\frac{2\times P\times R}{P+R}$$

where P is the accuracy of the model, and R is the recall rate.

## 4. Experiment

### 4.1. Model and Layout Settings and Data Acquisition

**Figure 4:**
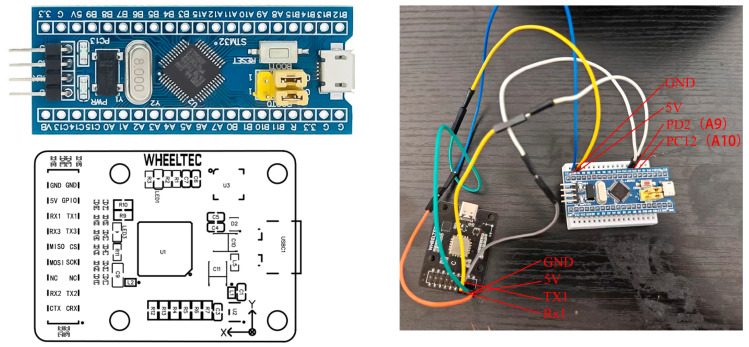
Schematic diagram of equipment construction.

In this research, to ensure greater stability for more accurate data collection, the design of the wearable device is divided into two parts: the internal structure must guarantee the stability of the prototype, while the exterior should be lightweight and simple, facilitating easy wear and portability in emergencies. Based on this core concept, the exterior design of the wearable device is developed. First, extract the shapes of the circuit board and breadboard, and arrange them to occupy the minimum space as shown. The inertial navigation module uses serial communication as its default communication method. Serial communication is a type of computer communication method, primarily serving to transfer data between the host and peripherals. It sends and receives bytes bit by bit. Its communication principle is simple and can achieve long-distance communication. The serial connection method for the inertial navigation module is shown in Figure 4.

PD2(A9)—Inertial Navigation Module Tx1

PC12(A10)—Inertial Navigation Module Rx1

GND—GND

5 V—5 V

Firstly, the design style is intended to be simple, easy to wear, and convey a certain sense of technology. The overall design ensures stability when worn. Secondly, in terms of cost, it is well controlled to enhance market competitiveness. As analyzed in the previous chapter, the overall layout tends to be rectangular. From a physical standpoint, a rectangle offers high space utilization, a stable structure, easy integration, and relatively simple manufacturing. From an aesthetic perspective, the symmetry and right angles of a rectangle convey a sense of balance and order, overall aligning well with the design requirements of this wearable device model. The use of straps as a wearing method can further improve the wearer’s comfort and enhance the user’s wearing experience.

**Figure 5:**
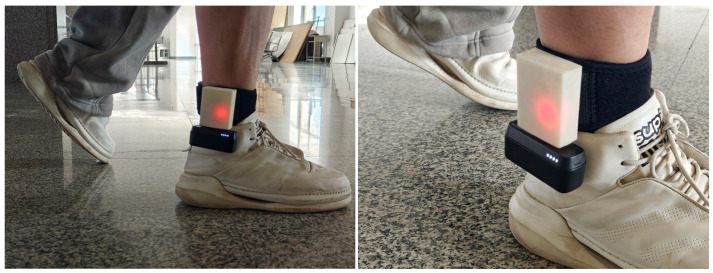
Schematic diagram of IMU installation.

The IMU was placed on the foot of the pedestrian. The installation method is shown in Figure 5, and the IMU was fixed on the outer side of the right foot.

The movement state data of people with different physical characteristics were collected and used as a test set and a training set, which included walking, fast walking, stair ascent, and stair descent data. The data of the training set are shown in Table 2, and the data of the test set are shown in Table 3.

### 4.2. Data Preprocessing

In this research, the researchers first obtained six-dimensional inertial measurements from gyroscopes and accelerometers. The feature labels for different motion states were itemized. The 3C method is based on fixed thresholds to improve the adaptability of the pedestrian navigation system between different gait states. It is also necessary to improve the accuracy of the feature labels of the training set as much as possible by continuously adjusting the thresholds of the 3C conditions.

When selecting ZUPT intervals, the researchers adopted Li Chao et al.’s method [14] to address the appearance of small burr signals. To solve this problem, according to the median filtering technique adopted by Li Chao, we obtained a zero-velocity sequence that basically conformed to the zero-velocity detection. Due to the nature of climbing upstairs, climbing downstairs, and other motion states, the data collected by the IMU became more complex, and the 3C threshold based on the walking state was no longer applicable. Therefore, the thresholds of the 3C method for additive synthetic amplitude, additive local variance, and gyroscope synthetic amplitude all needed to be adjusted.

**Figure 6:**
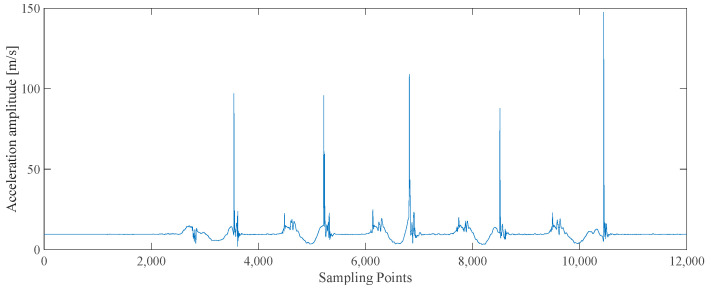
The acceleration amplitude graph.

**Figure 7:**
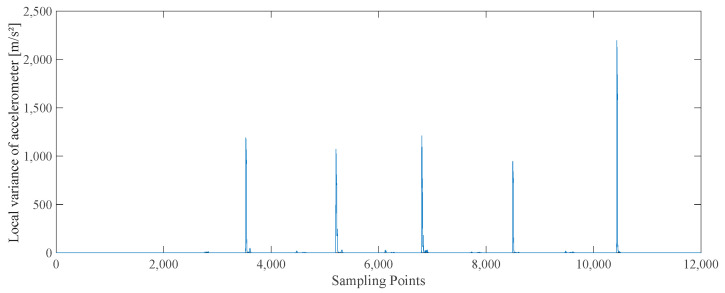
The local variance of accelerometer graph.

**Figure 8:**
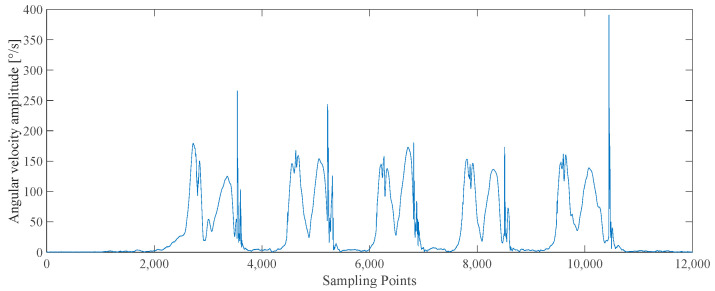
The angular velocity amplitude graph.

Taking the data of 10 steps of upward motion as an example, the acceleration amplitude, local variance of the accelerometer output, and angular velocity amplitude of the whole process are shown in Figure 6, Figure 7, and Figure 8, respectively.

**Figure 9:**
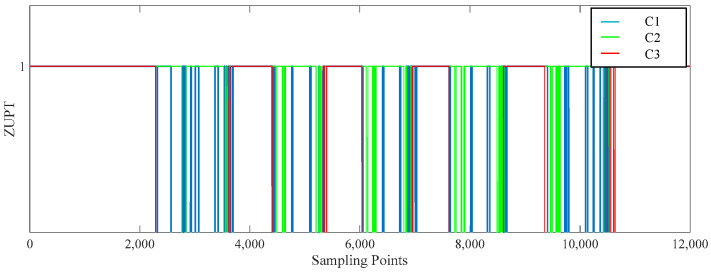
Stair ascent result graph. C1 represents the judgment of acceleration amplitude. C2 represents the judgment of acceleration variance, and C3 represents the judgment of angular velocity amplitude. (When the value of ZUPT is 1, the pedestrian movement is stationary; when the value of ZUPT is 0, the pedestrian movement is in motion).

Since this research employs a single foot sensor, there were five stationary times throughout the process, and the results of climbing upstairs in each of the 3C conditions are shown in Figure 9.

**Figure 10:**
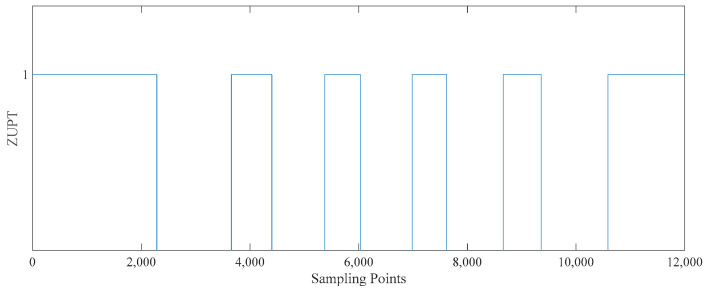
The ‘3C’ result graph. When the value of ZUPT is 1, the pedestrian movement is stationary. When the value of ZUPT is 0, the pedestrian movement is in motion.

There are more burr signals after the three ‘and’ conditions. The ZUPT sequence after the ‘and’ condition and median filtering is shown in Figure 10.

**Figure 11:**
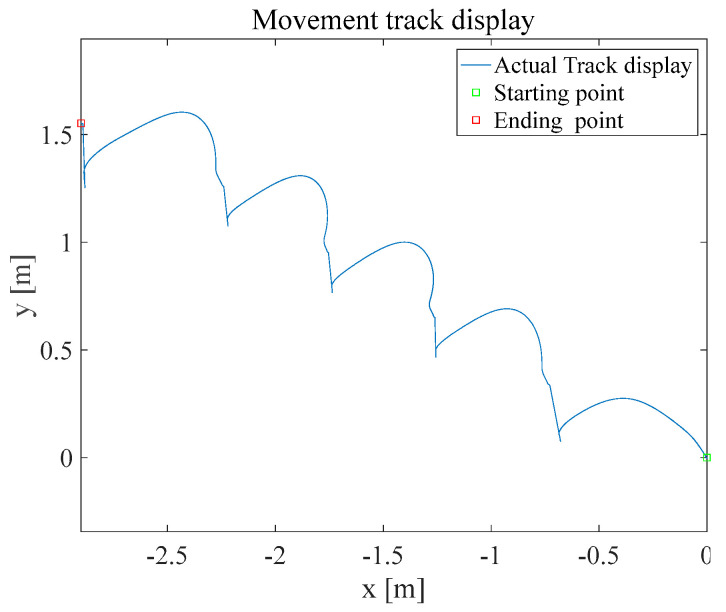
ZUPT navigation trajectory replication diagram for ‘3C’ condition.

The trajectory of climbing upstairs in the navigation is shown in Figure 11.

Similarly, this research made an optimization for the 3C method of climbing downstairs and fast walking, which could identify the ZUPT intervals of the movement states.

Finally, this research combined the above ZUPT tag sequences of different motion states with the six-dimensional inertial measurement data, thus obtaining the seven-dimensional data needed for the Informer neural network.

### 4.3. Model Training Process

The total mileage of the training and test sets were 3160 m and 2610 m, respectively. The input sequences of the model included six-dimensional inertial measurement data and one-dimensional labeled data.

**Figure 12:**
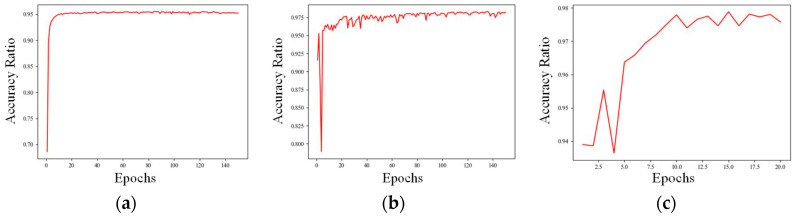
Accuracy result graph for each model, the red curve represents the test set accuracy of the models. (**a**) is the accuracy curve of the CNN model; (**b**) is the accuracy curve of the CNN + LSTM model; (**c**) is the accuracy curve of the Informer model.

Finally, to verify the performance of the Informer model, a CNN model and CNN + LSTM model were introduced in the model training part of this research as a comparison test. A 2000 m long sequence was used as the test set, and the red curve represents the test set accuracy of the models. The CNN model and the CNN + LSTM model were set to 150 rounds of training, and the Informer model was set to 20 rounds of training. The training set accuracy of the CNN model converged to around 95%. The results are shown in Figure 12.

The F1-scores of the three models in different test sets are shown in Table 4.

### 4.4. Analysis of Navigation Results

Because of the huge amount of time and long distance required for rescuers in sheltered spaces to carry out a rescue, this research simulated a 2000 m long-distance walking test on the ground with an experimental sampling number of 155,2906 and a sampling time of 1/1000 s.

**Figure 13:**
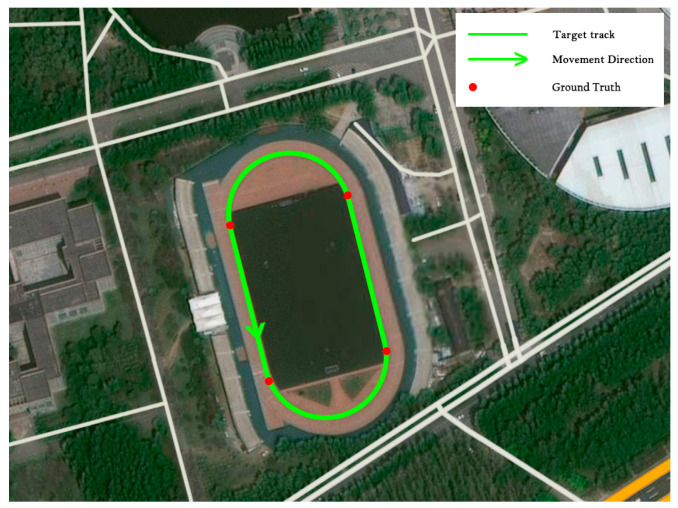
Target trajectory map.

To verify the performance of the proposed algorithm, a comparison test was conducted with the help of the zero-velocity update algorithm based on the CNN model and the zero-velocity update algorithm based on the CNN + LSTM model. The participants of this research were required to walk around a standard 400-m track at the selected site. Each participant walked five laps counterclockwise on the sports field along the trajectory of the innermost lane of the track. The starting and ending points of the straight path were taken as the true value points. The satellite map is shown in Figure 13.

The coordinates of the ground truth points in the x–y coordinate system are shown in Table 5.

**Figure 14:**
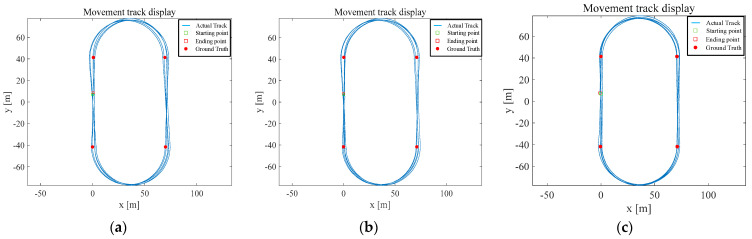
The solution trajectory diagram for each model. (**a**) is the trajectory of the CNN model; (**b**) is the trajectory of the CNN + LSTM model; (**c**) is the trajectory of the Informer model.

For the 2000 m motion experimental data, the CNN model, CNN + LSTM model, and Informer model were used for the prediction of the ZUPT interval. Zero-velocity correction was performed in the Kalman filter model with the starting position coordinates of (0,7), and the final solution trajectories of each model for the 2000 m motion model are shown in Figure 14.

**Figure 15:**
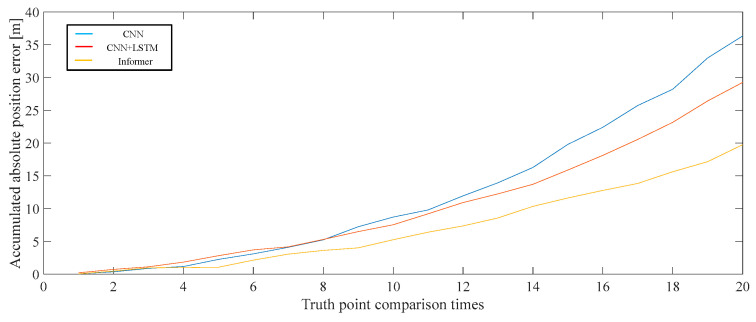
Cumulative error graphs for each model position.

The closed-loop error of the CNN model was 1.6 m, the closed-loop error of the CNN + LSTM was 1.132 m, and the closed-loop error of the Informer model was 1.136 m. The motion trajectory was cycled counterclockwise through the first, second, third, and fourth truth points five times. The positioning error was measured 20 times. The total of 20 accumulated position errors is shown in Figure 15.

In the walking experiment at 2000 m, all three types of models were able to identify the output zero-velocity motion state, because the noise of the gyroscope and accelerometer was relatively small. Similarly, the accuracy of both the CNN + LSTM model and the Informer model was better than the CNN model. The cumulative localization error of 20 truth points of the CNN model was 36.33 m, the cumulative localization error of the CNN + LSTM model was 29.26 m, and the cumulative localization error of the Informer model was 19.77 m.

**Figure 16:**
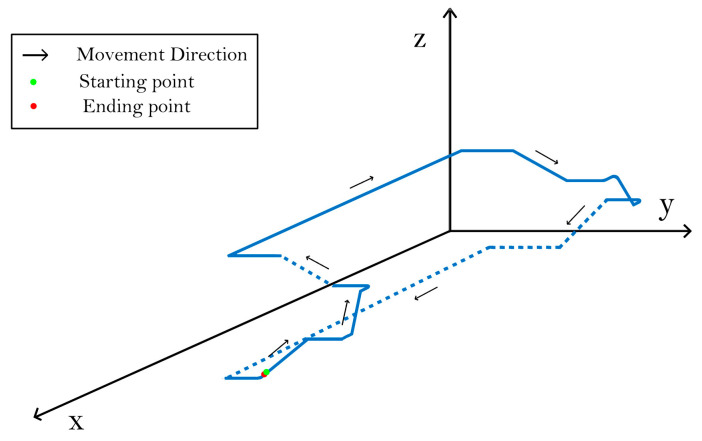
Diagram of mixed motion trajectory. Both solid lines and dashed lines represent motion trajectories. The dashed lines indicate the perspective structure in the three-dimensional view.

For the movement experiment of the underground space, due to the existence of height variation, this research designed a mixed-gait experimental test with significant height variation, in which the performance of the model in walking, climbing upstairs, and climbing downstairs was verified. The participant took the stairs of the third floor as the starting point, passed through the corridor of the fourth floor, reached the stairs on the other side, and then returned to the starting point of the third floor via the stairs. The schematic diagram of the motion trajectory is shown in Figure 16.

**Figure 17:**
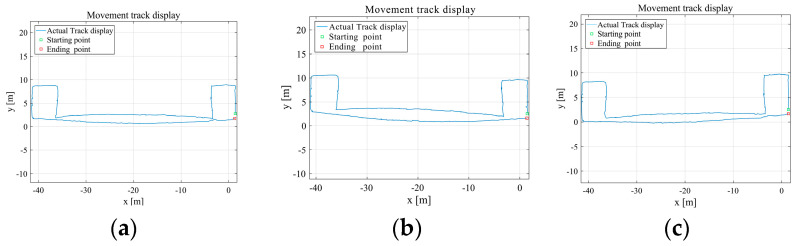
Navigation solution trajectories of the three models in the x–y coordinate system.

**Figure 18:**
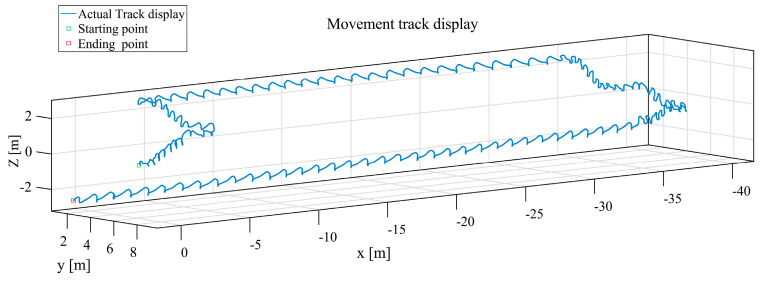
Navigation solution diagram for 3D coordinate CNN model, the blue trajectory line represents the inertial navigation trajectory solution calculated using a CNN model.

**Figure 19:**
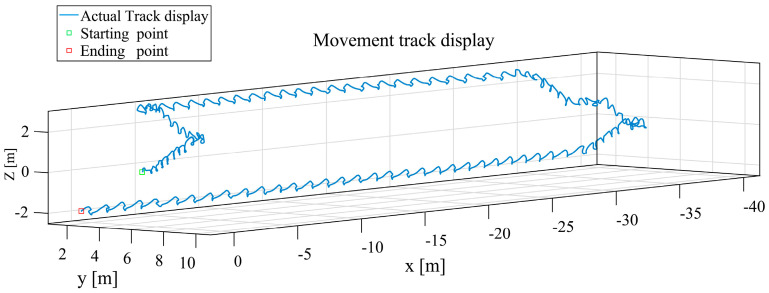
Navigation solution diagram for 3D coordinate CNN + LSTM model, the blue trajectory line represents the inertial navigation trajectory solution calculated using a CNN + LSTM model.

**Figure 20:**
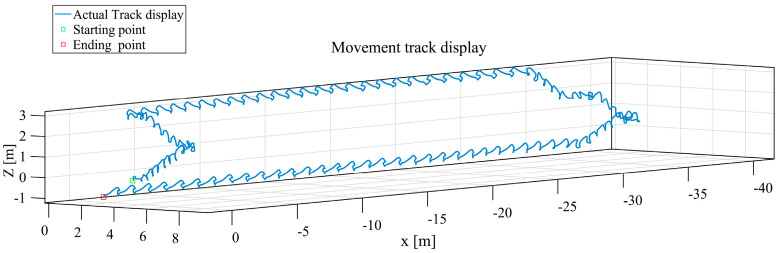
Navigation solution diagram for 3D coordinate Informer model, the blue trajectory line represents the inertial navigation trajectory solution calculated using a Informer model.

The results of the navigation solution trajectories of the three models in the x–y coordinate system are shown in Figure 17.

(Figure 17a) The results of the navigation solution of the CNN model in the 3D coordinate system, are shown in Figure 18.

(Figure 17b) The results of the navigation solution of the CNN + LSTM model in the 3D coordinate system are shown in Figure 19.

(Figure 17c) The results of the navigation solution of the Informer model in the 3D coordinate system are shown in Figure 20.

Based on the navigation trajectory, each model could effectively identify each motion state. The traditional ZUPT method could only suppress the error dispersion in the plane coordinate system, but not the error in height. Therefore, the error in height was mostly compensated for by the barometer. In addition, the neural network-based model could suppress the error dispersion on the z-axis to some extent. Because there are differences between the network models, the errors that led to the termination point in the z-axis direction varied. The z-axis direction error of the CNN model was 2.913 m, the z-axis direction error of the CNN + LSTM model was 2.277 m, and the z-axis direction error of the Informer model was 1.5052 m. Compared with the CNN model and the CNN + LSTM model, the Informer model could effectively suppress the divergence in height while adapting to different motion states.

## 5. Wearable Device Design Scheme

### Specific Analysis of the Design

This research proposed an improved Informer neural network zero-speed update algorithm for pedestrian navigation, which could not only adaptively predict the zero-velocity state of rescuers in different motion states through feature labeling of the original data and training, but could also overcome the problem of poor adaptability of the fixed-threshold PDR system. Finally, by constructing the Kalman filter, the pedestrian navigation system that was suitable for underground or sheltered spaces was obtained. The zero-velocity update architecture proposed in this paper reduced the error in height by 63.6% compared to the CNN model and by 53.6% compared to the CNN + LSTM model.

The CNN model had a limited ability to model time-series data. The improved CNN + LSTM model was based on the CNN model with the introduction of the LSTM layer, which could better model time-series data. The Informer model had a similar accuracy to the CNN-LSTM model. However, compared with the traditional CNN model and CNN-LSTM model, which can only extract information in local regions and cannot model long sequences well, the Informer model uses a self-attention mechanism to capture long-range dependencies in sequence data and better identifies the motion states in pedestrian navigation.

The main contributions of this paper are as follows:

A zero-velocity update architecture for pedestrian navigation based on an improved Informer neural network was proposed, which can be better applied to foot PDR systems to overcome the problem of poor adaptability of fixed-threshold PDR systems, and the model was ultimately able to adaptively output the zero-velocity state of pedestrians during walking, stair ascent, and stair descent. The architecture could effectively identify the zero-velocity state of multiple motion states.

The test data collected in this paper contained 2000 m of walking-gait data and 210 m of mixed-gait data. This paper compared the Informer model with a CNN model and CNN-LSTM model. Furthermore, this research successfully verified that the pedestrian zero-velocity update architecture based on the Informer model could be better applied to the environment of underground or obscured spaces without relying on external sources of navigation information.

In addition, this approach requires a large amount of training data and computational resources. In the future, the researchers will continue to enrich the training set data to reach the goal of achieving more motion state recognitions of pedestrians.

## Figures and Tables

**Table 1:** Zero offset of accelerometer and gyroscope.

Zero Offset	x-Axis	y-Axis	z-Axis
Accelerometer (mg)	−35.2524	1.0036	19.4669
Gyroscope (°/s)	−0.0412	−0.0396	−0.0051

**Table 2:** The training set data composition.

Sport Category	Distance (m)	Collection Times	No. of People	Average Number of Samples
Walk	200	10	5	156,652
Fast walk	100	10	5	76,314
Stair ascent	8	10	5	12,533
Stair descent	8	10	5	10,511

**Table 3:** The test set data composition.

Sport Category	Distance (m)	Collection Times	No. of People	Average Number of Samples
Long-distance walk	2000	1	1	155,2906
Hybrid walk	210	1	1	215,425

**Table 4:** The F1-scores of the three models.

F1-Score (%)	CNN	CNN + LSTM	Informer
Long-distance walk	96	97	98
Hybrid walk	96	98	98

**Table 5:** The ground truth point coordinates.

Coordinate Axis	Point No. 1	Point No. 2	Point No. 3	Point No. 4
x	0	72	72	0
y	−42.195	−42.195	42.195	42.195

## Data Availability

The dataset provided in this paper is not easily accessible, as it is part of an ongoing research study. Due to technical, temporal, and on-site objective limitations, the application scenarios of the dataset are restricted. Requests for access to the dataset should be sent directly to the email address zhangshuaib401@sylu.edu.cn.

## Abbreviations

The following abbreviations are used in this manuscript.

GNSS	Global Navigation Satellite System
UWB	Ultra-Wide-Band
ZUPT	Zero-Velocity Update
ZARU	Zero-Angular-Rate Update
AFM	Adaptive Fusion Module
ESKF	Error-State Kalman Filter
IMU	Inertial Measurement Unit
PDR	Pedestrian Dead Reckoning
CNN	Convolutional Neural Network
LSTM	Long Short-Term Memory
